# Study of endothelial function response to exercise training in hypertensive individuals (SEFRET): study protocol for a randomized controlled trial

**DOI:** 10.1186/s13063-016-1210-y

**Published:** 2016-02-13

**Authors:** Marinei Lopes Pedralli, Gustavo Waclawovsky, Augusto Camacho, Melissa Medeiros Markoski, Iran Castro, Alexandre Machado Lehnen

**Affiliations:** Laboratório de Investigação Clínica, Instituto de Cardiologia / Fundação Universitária de Cardiologia – ICFUC, Av. Princesa Isabel, 395. Bairro Santana, 90620 001 Porto Alegre, RS Brazil; Universidade Luterana do Brasil – ULBRA, Torres, Rio Grande do Sul Brazil; Faculdade Sogipa de Educação Física, Porto Alegre, Rio Grande do Sul Brazil

**Keywords:** Endothelial function, Exercise training, Hypertension

## Abstract

**Background:**

Endothelial dysfunction is a hallmark of diabetes mellitus and systemic arterial hypertension (SAH) and an early maker for atherosclerosis. Aerobic exercise training is known to enhance endothelial function, but little is understood about the effects of resistance or combined exercise training on endothelial function. The aim of this study is to investigate the effect of a 12-week aerobic (AT), resistance (RT), or combined (aerobic and resistance, CT) training program on endothelial function and assess associated effects on blood pressure in individuals with SAH.

**Methods/design:**

Eighty-one subjects with SAH aged 18 to 70 years will be selected and randomly assigned to three types of exercise training: AT, RT or CT. The study will involve the following procedures and tests: anamnesis, anthropometric assessment, echocardiography, blood pressure measurements through ambulatory blood pressure monitoring, flow-mediated dilation, ergospirometry, one repetition maximum test (1-RM), and blood collection (number of circulating endothelial progenitor cells, number of circulating endothelial microparticles, lipid profile, glucose, glycated hemoglobin, and creatinine). The AT intervention will consist of a 40-min exercise session with progressive intensities ranging from 50 to 75 % of heart rate reserve. The RT intervention will consist of a 40-minute session with four sets of six to 12 repetitions with a rest period of 60 to 90 seconds between each set and each type of exercise. Weight loads will be adjusted to 60 to 80 % of 1-RM for six types of exercise. The CT intervention will consist of a 20-min aerobic exercise session, followed by an additional 20-min resistance exercise session; each resistance exercise will have two sets less to match the total training volume.

**Discussion:**

The study results are expected evidence of cardiovascular protective effects of different types of exercise training through the modulation of endothelial function in hypertensive individuals. Knowing the magnitude of improvement of endothelium-dependent vasodilation for the different types of exercise training can provide scientific evidence for the prescription of exercise programs for vascular protection targeting hypertensive individuals.

**Trial registration:**

The Brazilian Clinical Trials Registry (http://www.ensaiosclinicos.gov.br/) under RBR-9ygmdn and dated 1 March 2015.

## Background

Cardiovascular diseases are largely determined by multifactorial risk factors that tend to interact and may be present over the course of an individual’s lifetime. Obesity [1, 2], physical inactivity [3, 4], and insulin resistance [5] are all risk factors closely associated with systemic arterial hypertension (SAH) and diabetes mellitus type 2. In addition to these risk factors, smoking [6] and excess weight [7] contribute to endothelial dysfunction [8, 9], which is a hallmark of diabetes mellitus [10] and SAH [11] and an early maker for atherosclerosis [12].

The endothelium is a single layer of cells that lines the tunica intima of blood vessels [13] and plays a major role in the modulation of vascular angiogenesis, inflammatory responses, and vascular tone and permeability [14]. Vascular protection is particularly attributable to endothelial nitric oxide synthase (eNOS), an enzyme involved in the production of nitric oxide (NO).

The balance between endothelial injury and recovery is critical to continuously maintaining endothelial function [15]. The bone marrow releases a progenitor cell subtype called endothelial progenitor cells (EPCs), which have the ability to migrate to the peripheral circulation and differentiate into mature endothelial cells to maintain vascular integrity [16]. EPC levels have been associated with cardiovascular risk factors [17], endothelium-dependent vasodilation [18], and clinical outcomes, including hospitalization and cardiovascular death rates [19]. In patients with cardiovascular risk factors such as SAH [20], smoking [21], metabolic syndrome [22] and diabetes mellitus [23], circulating levels of EPCs can be reduced compared with healthy individuals of matching age and sex. Endothelial dysfunction has been associated with a decreased number and impaired function of EPCs in patients with SAH, diabetes mellitus, and metabolic syndrome, and thus an increased risk of atherosclerosis would be expected [24].

On the other hand, regular exercise, also known as exercise training, produces effects on the cardiovascular system and has been proven to restore endothelial function even in the presence of cardiovascular disease [25]. Regular exercise promotes changes in cholesterol levels and its subfractions; accelerates the removal of chylomicrons and low-density lipoproteins from circulation; increases the release of NO that seems to generate increased expression of eNOS [26]; increases the number of circulating EPCs in healthy individuals [27], as well as in individuals with established cardiovascular disease [28]; and improves plasma glucose [29], which has a direct link to impaired endothelial function [30]. This assumption is consistent with findings in healthy individuals [31], individuals with diabetes mellitus type 2 [32], and patients with cardiovascular conditions [33], showing that exercise training improved vascular function and overall health.

Evidence with clinical relevance exists on the impact of aerobic exercise on BP and blood glucose levels, but no consensus exists regarding the effects of resistance training and even less evidence is available on combined training (resistance and aerobic) and the regulatory mechanisms of endothelial function and its relationship with BP in hypertensive individuals. Thus, despite present evidence showing the benefits of exercise and physical training on blood pressure [34] and blood glucose levels [35], more studies are needed to investigate further the mechanisms involved with endothelial function especially their relationship with blood pressure reduction.

The objective of this study is to evaluate the chronic effects of exercise training on endothelial function modulation in hypertensive patients undergoing a 12-week exercise intervention of aerobic, resistance, or combined (aerobic and resistance) training. Modulation of endothelial function will be assessed by flow-mediated dilation (FMD) [36] and biochemical [37] and cellular parameters [38, 39]. The primary and secondary study objectives are listed in Tables 1 and 2, respectively.

**Table 1:** Primary objectives of the study

• To investigate the effects of AT, RT and CT on flow-mediated vasodilation of the brachial artery in hypertensive individuals • To assess the impact of different types of training (AT, RT, and CT) on the number of circulating endothelial progenitor cells and number of circulating endothelial microparticles in hypertensive individuals	

AT, aerobic training; RT, resistance training; CT, concurrent training

**Table 2:** Secondary objectives of the study

To assess the effectiveness of different types of training (AT, RT and CT) regarding the following:	
• Blood pressure levels: clinical assessment and ABPM • Serum levels of total cholesterol, HDL, LDL and triglycerides • Fasting blood glucose and glycated hemoglobin • Blood viscosity, RBC deformability, RBC aggregation, fibrinogen levels and ESR • Echocardiography for determination of heart chamber sizes and wall thickness • Functional capacity through maximum oxygen consumption (VO_2_max) • 1-RM • Abdominal obesity and BMI	

ABPM, ambulatory blood pressure monitoring; AT, aerobic training; RT, resistance training; CT, concurrent training; HDL, high-density cholesterol; LDL, low-density cholesterol; RBC red blood cell; ESR, erythrocyte sedimentation rate; 1-RM, one-repetition maximum strength test; BMI, body mass index.

## Methods/design

The study will be coordinated at the Fundação Universitária de Cardiologia (FUC) Health Sciences Graduate Program together with the Instituto de Cardiologia (IC) in the city of Porto Alegre, Rio Grande do Sul, Brazil.

### Study design

**Fig. 1:**
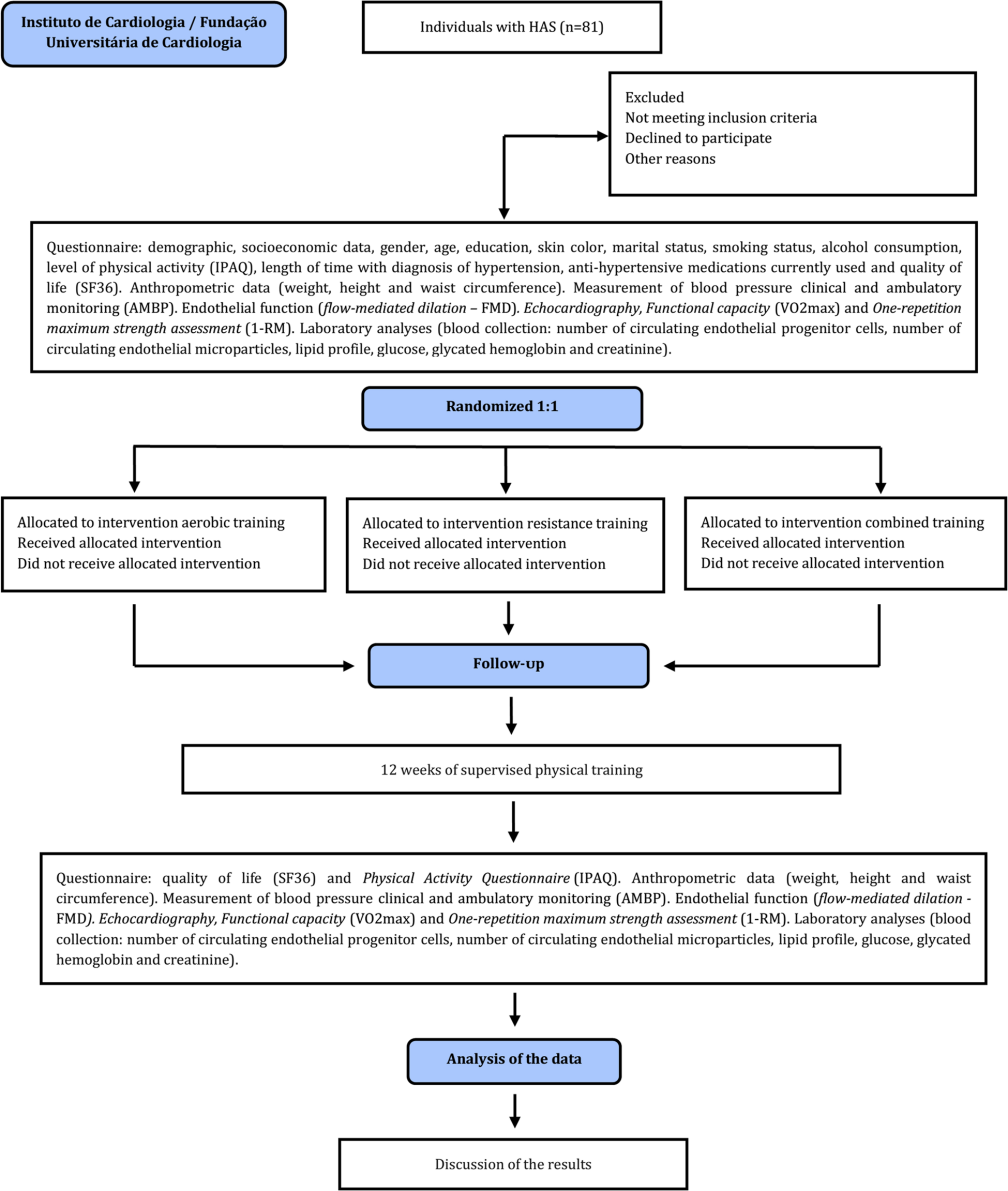
Study design. Flow chart of how the clinical trial will be conducted

A randomized controlled and evaluator-blinded clinical trial will be conducted as shown in Fig. 1.

### Sample selection

The study sample will comprise individuals with no history of cardiovascular disease (except for SAH controlled with the use of medications), neuromuscular, endocrine, and/or metabolic conditions (except for diabetes mellitus type 2) who are attending the IC health care facilities.

Subjects will be selected based on information collected from their medical records. Eligible individuals will be contacted by telephone and informed of the purpose and procedures of the study. For those willing to participate, a first visit will be scheduled when a clinical questionnaire will be administered, and they will be asked to sign a free informed consent form to participate in the study. Other study visits will be scheduled for those meeting the inclusion criteria of the study (Table 3).

**Table 3:** Inclusion and exclusion criteria for the study

Inclusion criteria	Exclusion criteria
• Males and females aged 18 to 70 years • Diagnosed with SAH • Signed a free informed consent form • Considered insufficiently active (IPAQ) • Non-active smokers	• Currently engaging in any type of systematic physical activity • Blood pressure hyper-reactivity in the clinical assessment (SBP > 220 mmHg and/or increase in DBP > 15 mmHg) • Ongoing infectious diseases • Obesity class II or III (BMI) • Heart failure class III or IV; recent cardiovascular event (in the last three months); chronic renal failure; and malignant disease with life expectancy < 2 years • Orthopedic impairments or any physical or mental limitations that prevent physical exercises • After the training intervention, individuals who attended < 85 % or missed > 2 consecutive training sessions will be excluded from the sample. Subjects will be encouraged to complete any missed session at the end of training by performing additional sessions.

SAH, systemic arterial hypertension; IPAQ, International Physical Activity Questionnaire; SBP, systolic blood pressure; DBP, diastolic blood pressure; BMI, body mass index

The following formula will be used for the calculation of sample size (n_p_) to estimate the number of hypertensive individuals to be included in the study:

$$ \left({\upmu}_{\mathrm{p}}\right):\ {\mathrm{n}}_{\mathrm{p}0} = {\mathrm{s}}^2/\ {\left({\upmu}_{\mathrm{p}}\hbox{-}\ {\mathrm{x}}_{\mathrm{p}}\right)}^2\mathrm{x}\ {\left({\mathrm{t}}_{\upalpha; \mathrm{g}\mathrm{l}}\right)}^2, $$

where, n_po_ = sample size at time point zero, s^2^ = variance of the sampling distribution, and t_α;gl_ = sampling distribution according to a predetermined error probability (*P* ≤ 0.05) and degrees of freedom for n_po_.

The mean incidence rate of SAH in Brazil is 20 % of the population [40]. Considering the baseline value (n_0_ = 30); t_α;gl_ = t_0.05;29_ = 2.045; s^2^ = 0.05 (based on the maximum value of the coefficient of variation of endothelial vasodilation); and the maximum error of the estimate taken as the maximum variability in arterial diameter measures by two observers (0.02 %) [41], it was estimated that n_p1_ = 26.1 ≅ 26 hypertensive individuals. Thus, in the estimate of *n,* the approximation used considered n_p1_ = 26, gl = 23, and t_0.05;23_ = 2.069. The n_p1_ = 26.8 ≅ 27. The estimate of the sample size for the study was *n* = 80.3 ≅ 81 hypertensive individuals who will be randomly assigned to the three training interventions.

### Randomization procedure

A randomization for the proposed interventions will be generated by a computer program (www.randomization.org) including the distribution coded at a 1:1 ratio. The random assignment of subjects to training intervention groups will be into three blocks of 27. Subjects will be drawn up to 27 in each intervention group: aerobic training (AT), resistance training (RT), and combined training (CT, resistance and aerobic). All subjects will be blinded to intervention allocation and will have access to this information only after the baseline assessment. All evaluators of the study subjects will be also blinded to intervention allocation.

### Baseline assessment of subjects

After recruitment, the subjects will undergo baseline assessment to collect information on their main characteristics before intervention. A descriptive exploratory questionnaire will be administered to collect information on i) demographic and socioeconomic variables (gender, age, socioeconomic status, education, skin color and marital status); ii) behavioral variables (physical activity measured by the International Physical Activity Questionnaire (www.ipaq.ki.se), smoking status, and alcohol consumption); iii) anthropometric variables (body weight, height, and waist circumference); iv) clinical variables (BP and use of antihypertensive medication); and v) quality of life assessed by the Medical Outcomes Study 36-Item Short Form Health Survey (SF-36) [42]. Eligible individuals who are willing to participate will be instructed to fast for 8 h and come for a scheduled second visit at the study site. In this visit, subjects will have blood samples collected for blood testing, in addition to the assessment of FMD of the brachial artery and ambulatory blood pressure monitoring (ABPM) cuff placement. A snack will be offered at the end of this visit. In the third visit, the ABPM cuff will be removed and subjects will undergo physical fitness tests (VO_2_max). In the fourth visit, 1-RM testing will be conducted. Subjects who did not show up for the scheduled visits will be contacted again. All blood samples will be collected by a nurse technician. All assessment procedures will be supervised by the study investigators and ICFUC medical staff.

### Assessments

#### Blood collection

Blood collection and testing will be performed at the ICFUC. Subjects’ blood will be collected after a fast of 8 h. The blood sample will be tested for biochemical screening (lipid profile, glucose, glycated hemoglobin, and creatinine), number of EPCs and number of circulating endothelial microparticles. In addition, blood rheology will be assessed for potential changes in the sample studied including increased blood viscosity, reduced red blood cell (RBC) deformability, increased RBC aggregation, fibrinogen levels, and erythrocyte sedimentation rate (ESR).

### Progenitor cell and endothelial progenitor cell count

A 15-ml blood sample for the EPCs will be collected in a Sodium Heparin Vacutainer™ tube. Peripheral blood mononuclear cells (PBMC) will be isolated using Ficoll-Paque (GE Heathcare Life Sciences, Uppsala, Sweden). Progenitor cells (PCs) will be defined as CD34+/CD45dim, and EPCs as CD34+/KDR+/CD45dim. Briefly, blood samples will be diluted once with phosphate buffered saline (PBS) and layered onto Ficoll-Paque in 15-ml conical tubes (BD Biosciences, San Jose, CA, USA). Each tube will be centrifuged at 400 xg for 30 min (Eppendorf-5810, Hamburg, Germany), and the PBMCs at the interface will be collected. Cells will be washed once with RPMI-1640 medium (Gibco™, Vancouver, Canada); stained with 5 μl of antihuman-CD45 FITC, 5 μl of antihuman-CD34 PE (clone 6G12), and 8 ul of antihuman-KDR-Alexa Fluor 647 (BD Biosciences, San Jose CA, USA); and incubated in the dark for 30 min, followed by the addition of 500 μl of PBS until resuspension and acquisition in a flow cytometer (FACSCalibur, CellQuest software, BD Biosciences, San Diego, CA, USA). The total number of events recorded on the mononuclear cells gate was 200,000. The percentage of CD34+ cells was calculated based on the measured number of leukocytes (CD45+ cells) using the ISHAGE (International Society for Hematotherapy and Graft Engineering) gating strategy [43].

### Circulating endothelial microparticles count

A 5-ml blood sample for circulating endothelial microparticles will be collected in a Citrate Vacutainer™ tube. These samples will be analyzed for CD31+/CD41- and CD144+. Initially, the blood will be rendered to separate the platelet-rich plasma and the platelet-poor plasma. All blood samples will be subjected to a two-step protocol centrifugation (860 xg for 15 min at 4 °C, followed by 1700 xg for 5 min at 20 °C) before being stored at -80 °C. Phenotype analysis of the endothelial microparticles will be subsequently performed on samples thawed using a flow cytometer Fluorescence Activated Cell Analyser (FACScanto II da BD Biosciences, San Jose, CA, USA) and the results analyzed with the BD FACS Diva software (BD). For phenotyping of the endothelial microparticles, the following antibodies will be used: anti-CD31+ PE, anti-CD41+ PC7, and anti-CD144+ PE. Endothelial microparticles will be defined as elements with size > 0.1 and < 1.0 μm and showing surface markers CD31+/CD41- and CD144+ [44].

### Assessment of flow-mediated dilation (FMD)

Endothelial function will be measured noninvasively by using brachial artery ultrasound (EnVisor series, Philips Ultrasound, Bothell, WA, USA). This assessment will be performed with a 7 to 12 MHz linear transducer in a heated room at 21 to 24 °C at ICFUC. A software program will be used for two-dimensional color-Doppler imaging acquisition with a simultaneously recorded electrocardiogram (ECG). Image data will be recorded on a DVD. To minimize operational errors, the subject’s arm, and the ultrasound transducer will be positioned and maintained in the same position during the procedure. Baseline images will be recorded. A cuff will be placed around the forearm and inflated to 50 mmHg above systolic pressure for at least 5 min and then removed. Longitudinal ultrasound images of the brachial artery walls will be acquired to assess endothelium-dependent vasodilation. The expected coefficient of variation between the images will be 1.8 to 5.0 %. Flow-dependent vasodilation responses will be expressed as the percentage change in the brachial diameter [36].

### Echocardiography

Echocardiography is a noninvasive imaging approach that uses ultrasound to assess the structures and functioning of the heart; it measures the chamber size and mobility of the walls, valve structures, ejection fraction, diastolic dysfunction parameters, and the direction and speed of blood flowing through the heart chambers. Medical personnel at ICFUC accredited by the Brazilian Society of Echocardiography and blinded to intervention allocation will perform transthoracic echocardiograms with image acquisition in second-harmonic generation with the use of ACUSON SC2000 equipment and a 3 to 12 MHz transducer (Siemens). This data will be digitally stored for further analysis by more experienced evaluators.

### Blood pressure

Two different approaches will be used to evaluate the BP levels: clinical assessment and ambulatory blood pressure monitoring (AMBP). The clinical assessment will consist of acute and subacute BP measurements as a way to evaluate exercise responses. Measurements will be taken with the subject in a sitting position using a semi-automatic sphygmomanometer (Omron 705CP). BP levels will be measured in both arms in the pre-study assessments, and the arm with highest values will be used for measurements in the study. Subacute clinical assessments will be conducted at the following time points: before the beginning of each training session (pre-exercise), immediately after exercise (early post-exercise), and 20 min after the end of each session (late post-exercise). ABPM will be performed in all study groups before the first exercise session and at the completion of the entire training intervention. Blood pressure measurements will be obtained from an oscillometric Dina-map™ device. Both assessments will follow the VI Brazilian National Guidelines in Cardiology [40].

### Functional capacity

VO_2_max will be obtained from a cardiopulmonary exercise test using a cycle ergometer and 12-lead ECG monitoring supervised by a cardiologist. Gas exchange measurements will be taken with the use of an ergo spirometer (VO2000 model, Inbramed, Porto Alegre, Brazil). This device collects exhaled gas samples and can store data acquired with each breath (breath-by-breath). Heart rate will be monitored with a frequency meter (Polar S610 model) as follows: online heart rate readings, determination of maximum heart rate at each exercise stage, and stopwatch with partial times. The maximal exercise test will be scheduled at least 48 h in advance. Upon participant arrival at the study site, BP levels will be measured according to the study protocol. Subjects will be asked to sit on a chair, and a facemask (or nozzle) and a frequency meter will be attached. Resting VO_2_ and VCO_2_ measurements will be taken, and the test will be started only when the respiratory exchange ratio (RER) is lower than 0.95. The test will be stopped when the subjects are exhausted and signal that they can no longer turn the pedals [45].

### One-repetition maximum strength (1-RM) assessment

The one-repetition maximum strength test (1-RM) will be used to measure the maximum strength. Subjects will warm up on a cycle ergometer for 5 min. They will then be instructed to perform the maximum number of repetitions with a given load until they can perform only one lift of a load in up to five attempts to achieve the maximum load. A rest period of 3 to 5 min will be provided between each attempt, so rest intervals are of sufficient length to allow the restoration of energy reserves [46]. The same procedure will be followed for all types of strength exercises proposed in the study (leg press, bench press, knee extension, biceps curl, knee bend, and low row). The speed of movements will be controlled with a metronome in both intervention protocols. Exercises will be performed at a pace of 2 seconds for both concentric and eccentric phases. This test will take place at three time points: during the first 2 weeks (60 % of 1-RM); during the intermediate weeks (70 % of 1-RM); and at the end of week 8 (80 % of 1-RM) to maintain exercise at an appropriate intensity throughout the protocol.

### Intervention protocols

The study intervention will comprise 12 weeks of exercise sessions, lasting on average 50 min, three times a week. Exercise sessions will include a warm-up period of 5 to 10 min at the beginning, resistance or combined aerobic exercises for 40 min, and a cool down/relaxation period.

AT will be performed on a cycle ergometer for the entire intervention. Subjects will be monitored with heart rate monitors (Table 4).

**Table 4:** Intensity and duration of aerobic training on a stationary bike during a 12-week intervention

	Phase I	Phase II	Phase III
Intensity	50 to 65 % HRR	55 to 70 % HRR	60 to 75 % HRR
Session duration	40 min	40 min	40 min
Exercise duration (weeks)	4	4	4

HRR, heart rate reserve

RT will include six types of exercises that alternate between large and small muscle groups (leg press, bench press, knee extension, biceps curl, knee bend, and low row) for the entire training intervention (Table 5).

**Table 5:** Intensity, number of sets, repetitions, rest period between sets, and session duration of resistance training during a 12-week intervention

	Phase I	Phase II	Phase III
Intensity	60 % 1-RM	70 % 1-RM	80 % 1-RM
Sets	4	4	4
Repetitions	10 to 12	8 to 10	6 to 8
Rest period between sets	60 s	60 s	90 s
Session duration	40 min	40 min	40 min
Exercise duration (weeks)	4	4	4

1-RM, one-repetition maximum strength test

CT will consist of resistance and aerobic exercises combined in one session performed over a 40-min period set as in the other two protocols, so that training volume is similar across all intervention groups for the entire intervention (Table 6).

**Table 6:** Intensity, number of sets, repetitions, rest period between sets, and session duration of concurrent training during a 12-week intervention

	Phase I	Phase II	Phase III
Resistance training			
Intensity	60 % 1-RM	70 % 1-RM	80 % 1-RM
Sets	2	2	2
Repetitions	10 to 12	8 to 10	6 to 8
Rest period between sets	60 s	60 s	90 s
Session duration	20 min	20 min	20 min
Aerobic training			
Intensity	50 to 65 % HRR	55 to 70 % HRR	60 to 75 % HRR
Session duration	20 min	20 min	20 min
Exercise duration (weeks)	4	4	4

1-RM, one-repetition maximum strength test; HRR, heart rate reserve

A basic training principle is the progression of exercise intensity, duration, or frequency. Progression of exercise in the study intervention will be through gradual exertion increase for the protocols [46]. Borg’s rating scale of perceived exertion (6 to 20 point scale) will be used to help control the training intensities [47]. All subjects will be allowed an initial adjustment period. Those with resting BP levels higher than 160 mmHg (SBP) and/or 105 mmHg (DBP) before cardiopulmonary testing, 1-RM, or an exercise session (AT, RT and CT) will not be allowed to participate in any activities [40]. Those experiencing BP higher than the recommended level on a second occasion will be referred to the study cardiologist for evaluation. The cardiologist will determine whether the subject can continue participating in the study.

Subjects diagnosed with SAH and diabetes mellitus type 2 will have their blood glucose checked (Optium Xceed, Abbott Diabetes Care, Inc., Alameda, CA, USA) in addition to BP measurements before cardiopulmonary exercise testing, 1-RM or an exercise session (AT, RT, and CT) and every 15 min throughout exercise sessions. If the blood glucose levels are lower than 100 mg/dL during cardiopulmonary exercise testing, 1-RM, or exercise sessions (AT, RT and CT), subjects will receive 30 to 45 g glucose gel (Gli-Instan, Lightsweet ™) to maintain levels above 120 mg/dL, and their blood glucose will be checked again 15 min later. Those with blood glucose higher than 250 mg/dL will have their urine tested for the presence of ketones (ComboStik™, Gyung-Nam, Korea), and they will be allowed to resume exercise only if test results are negative. Subjects with blood glucose lower than 100 mg/dL during exercise sessions will have to discontinue exercising and be offered a snack. Their blood glucose will be tested again 15 min later, and they will be allowed to resume exercise only if their test results are acceptable [48].

### Statistical procedures

For data comparison between pre-exercise and post-exercise and across groups, nonparametric (Spearman’s correlation (ρ), chi-square test (χ^2^), and Mann–Whitney U test), and parametric tests (t-test, analysis of variance (ANOVA), multivariate analysis of variance (MANOVA), multivariate linear regression, and canonical discriminant analysis) will be used depending on the variable studied, as well as *post hoc* tests (Duncan and Waller-Duncan - ANOVA and MANOVA) [49]. The significance level will be set at 0.05. All statistical analysis will be performed using SPSS version 22.0.

### Ethical issues

The study protocol followed the principles of the Declaration of Helsinki and was approved by the research ethics committee (Protocol 925.406 by Instituto de Cardiologia do RS/Fundação Universitária de Cardiologia – Brazil, dated 22 December 2014 and signed by Dr. Leonardo Martins Pires). Furthermore, all subjects will read and give their informed consent for participation in the research study. All information will be confidential, subjects’ names will be kept confidential, and data will be collected for academic purposes only according to Resolution CNS N1466/12 [50]. If subjects in the RT and CT groups do not show any improvements of endothelial function (primary endpoint) or BP levels compared to those in the AT group, they will be offered the opportunity to practice the AT protocol after completion of the study to ensure equal benefits for all subjects.

## Discussion

The Study of Endothelial Function Response to Exercise Training (SEFRET) is a single-site study that will be conducted in the city of Porto Alegre, Brazil. The study will be conducted in accordance with the principles of Good Clinical Practice and all requirements established by ICFUC relevant bodies and collaborators.

The study results are expected to provide evidence of cardiovascular protective effects of different types of exercise training by modulating endothelial function in individuals with SAH. Knowing the magnitude of improvement of endothelium-dependent vasodilation by different types of exercise training can provide scientific evidence to the prescription of exercise programs for vascular protection targeted to hypertensive individuals. An adequate exercise program can lead to significant changes in cardiovascular function and promote health benefits and BP control, thus reducing adverse cardiovascular outcomes.

### Trial status report

Recruiting.

## Abbreviations

ABPM: ambulatory blood pressure monitoring
AT: aerobic training
BMI: body mass index
BP: pressure blood
CT: combined training
DBP: diastolic blood pressure
ECG: electrocardiogram
eNOS: endothelial nitric oxide synthase
ESR: erythrocyte sedimentation rate
FMD: flow-mediated dilation
FUC: Fundação Universitária de Cardiologia
HDL: high-density cholesterol
HRR: heart rate reserve
IC: Instituto de Cardiologia
IPAQ: International Physical Activity Questionnaire
LDL: low-density cholesterol
NO: nitric oxide
PBMN: Peripheral blood mononuclear cells
PBS: phosphate buffered saline
PCs: Progenitor cells
RBC: red blood cell
RT: resistance training
SAH: systemic arterial hypertension
SBP: systolic blood pressure
WHO: World Health Organization
1-RM: one repetition maximum.
